# Clinico-pathological correlations of congenital and infantile nephrotic syndrome over twenty years

**DOI:** 10.1007/s00467-014-2856-x

**Published:** 2014-06-06

**Authors:** Jameela A. Kari, Giovanni Montini, Detlef Bockenhauer, Eileen Brennan, Lesley Rees, Richard S. Trompeter, Kjell Tullus, William van’t Hoff, Aoife Waters, Emma Ashton, Nicholas Lench, Neil J. Sebire, Stephen D. Marks

**Affiliations:** 1 Department of Paediatric Nephrology, Great Ormond Street Hospital for Children NHS Foundation Trust, Great Ormond Street, London, WC1N 3JH UK; 2 Department of Pathology, Great Ormond Street Hospital for Children NHS Trust, London, WC1N 3JH UK; 3 NE Thames Regional Genetics Service, Great Ormond Street Hospital for Children NHS Trust, London, WC1N 3JH UK; 4 Department of Paediatrics, Faculty of Medicine, King Abdulaziz University, Jeddah, Kingdom of Saudi Arabia; 5 Pediatric Nephrology Unit, Department of Pediatrics, Azienda Ospedaliero-Universitaria Sant’Orsola-Malpighi, Bologna, Italy

**Keywords:** Congenital nephrotic syndrome, Infantile nephrotic syndrome, Histopathology, Genetics, Outcome

## Abstract

**Background:**

Nephrotic syndrome (NS) presenting early in life is caused by heterogeneous glomerular diseases. We retrospectively evaluated whether histological diagnosis in children presenting with NS in the first year of life predicts remission or progression to end-stage kidney disease (ESKD).

**Methods:**

This is a single centre retrospective review of all children diagnosed with NS before one year of age between 1990 and 2009. All subjects had a renal biopsy, which was independently blindly reviewed by a single renal pathologist for the purpose of this study.

**Results:**

Forty-nine children (25 female) who presented at 0.1–11.6 (median 1.6) months were included with 31 presenting within the first three months of life. Histopathological review diagnostic categories were; 13 Mesangial proliferative glomerulopathy (MesGN), 12 Focal and segmental glomerulosclerosis (FSGS), 11 Finnish type changes, eight Diffuse Mesangial Sclerosis (DMS), three Minimal change disease (MCD) and one each of Dense Deposit Disease (DDD) and Membranous nephropathy. Two children died from haemorrhagic complications of the biopsy. Eight children achieved remission (four MesGN, one Finnish type changes, one FSGS, one MCD and one membranous) with patient and renal survival of 73 % and 43 %, respectively, at follow-up duration of 5–222 (median 73) months (with five lost to follow-up). All children with Finnish-type histopathological changes presented within five months of age. Due to the historical nature of the cohort, genetic testing was only available for 14 children, nine of whom had an identifiable genetic basis (seven NPHS1, one PLCE1 and one ITGA3) with none of these nine children achieving remission. All of them had presented within four months of age and required renal replacement therapy, and two died.

**Conclusions:**

Histopathological findings are varied in children presenting with NS early in life. Whilst groups of histological patterns of disease are associated with differing outcomes, accurate prediction of disease course in a specific case is difficult and more widespread genetic testing may improve the understanding of this group of diseases and their optimal management

## Introduction

Nephrotic syndrome (NS) which appears early in life provides a diagnostic and management challenge to pediatric nephrologists[1]. It is caused by heterogeneous glomerular diseases, and the diagnosis is based on clinical, laboratory, and histological criteria[2]. Congenital nephrotic syndrome (CNS) is a term used for NS presenting within the first three months of age while infantile nephrotic syndrome (INS) is used for NS presenting between three and 12 months of age.

CNS and INS are commonly associated with Finnish type nephropathy [3] or Diffuse Mesangial Sclerosis (DMS) on histological examination[4, 5]. However, other glomerular pathologies have also been reported such as focal and segmental glomerulosclerosis (FSGS) and minimal change disease (MCD)[6, 7].

Early onset NS is usually caused by a genetic defect in a major podocyte slit diaphragm protein, Nephrin (NPHS1)[8], or less commonly by mutations in WT1[9], PLCE1 [10], LAMB2 [11] or NPHS2 [12]. Non-genetic causes include infections such as congenital toxoplasmosis [13], cytomegalovirus infection [14], or congenital syphilis [15]. Hinkes et al. [12] reported that two-thirds of nephrotic syndrome manifesting in the first year of life can be explained by mutations in four genes (NPHS1, NPHS2, WT1, or LAMB2), and these children with identified genetic causes should not be treated with corticosteroids, as they are steroid-resistant. In addition, there are children with congenital NS who spontaneously improve: some of these patients may have anti-glomerular antibodies transferred from the mother [16], although in many children, the reason for developing NS is unexplained[17].

A renal biopsy in children under 12 months of age with NS can, however, be technically difficult to perform, with potentially serious complications, especially in small oedematous children. The clinical utility of the biopsy result with respect to prognosis and management is unclear in the era of increased genetic testing. In the present study, we, therefore, retrospectively investigated the histological findings and clinical course of children who presented within one year of age with NS and required admission to the pediatric nephrology unit at Great Ormond Street Hospital for Children NHS Foundation Trust (GOSH) over twenty years between 1990 and 2009, and in whom a renal biopsy was performed. Ethical approval was obtained for this study from the University College London Institute of Child Health and Great Ormond Street Hospital for Children NHS Foundation Trust Research Ethics Committee.

## Patients and methods

Inclusion criteria were children who presented within the first year of life with nephrotic syndrome who had a histological diagnosis obtained by renal biopsy between January 1990 and December 2009. The clinical diagnosis of NS was made on the basis of nephrotic range proteinuria (urine albumin/creatinine ratio (UA:UC) above 200 mg/mmol), hypoalbuminaemia (<25 g/l) and oedema. Clinical management over the years before genetic testing was available usually included a renal biopsy in patients with CNS or INS unless clinical management with early nephrectomy was planned. However, this clinical practice was modified with priority given to genetic studies in recent years.

Clinical, laboratory, and histological data were collected from the medical notes of the patients, including age, renal function, and quantification of albuminuria at onset of the disease and at the time of renal biopsy. Data was recorded on family history (including parental consanguinity), antenatal history, birth weight, treatment [including immunosuppressive agents, angiotensin converting enzyme inhibitors (ACEi) and/or angiotensin receptor blockers (ARB)], clinical course [including remission of NS, unilateral or bilateral native nephrectomies, or requirement for end-stage kidney disease (ESKD) management with dialysis and/or transplantation] and long-term follow-up. Extra-renal manifestations (neurological, ophthalmological, hepatic, and gastro-intestinal involvement) were evaluated from the notes.

All but six percutaneous renal biopsies were performed at GOSH under ultrasound guidance and examined by light microscopy, immunohistochemistry and electron microscopy. The other children were referred with slides of renal biopsies, which were performed at other institutions. All renal histology was re-evaluated by a single pediatric renal pathologist (NS) for the purpose of this study who was blinded to the clinical outcomes of the patients. In addition, the histopathological findings of 13 children who had nephrectomies (ten bilateral and three unilateral) were compared with their initial renal biopsy findings.

## Results

### Baseline demographics

Forty-nine children (25 female) from 48 different families were included in the study. All had onset of NS within the first year of life with renal histology obtained. The age at presentation with NS was 0.1–11.6 (median 1.6) months, with 31 presenting within the first three months. Renal function varied at presentation, with plasma creatinine of 4–160 (median 35) μmol/l. There was a high rate of consanguinity, with 15 parents being first cousins or second cousins and seven children had affected siblings with CNS (Table 1).

**Table 1:** Demographic, biopsy data and response to treatment in different groups

	Number	Female	Age at presentation (months)	Age at first renal histology (months)	Number of glomeruli	UA: UC (mg/mmol) at presentation	Family history	Parental consanguinity	Gestational age (range)	Steroid treatment	ACE inhibitors or ARBs
MesGN	13	5(38.5 %)	0.8 (0.1-7.2)	5.5 (1-8)	20 (5-105)	2600 (460-20400)	3(23.1 %)	4(30.8 %)	39(34-40)	1 (7.7 %)	7(53.8 %)
**FSGS**	12	7 (58 %)	1.5 (0.1-9.9)	5 (2-16)	31 (13-56)	2910 (644-3444)	(0 %)	4 (40 %)	40 (35-40)	0 (0 %)	4 (80 %)
**Finnish**	11	7 (63.6 %)	1.0 (0.0-4.8)	7 (3-16)	27 (5-100)	5945 (2900-8460)	1(9.1 %)	4 (36.4 %)	35 (25-40)	1 (9.1 %)	4 (36.4 %)
**DMS**	8	2 (25 %)	4.7 (0.2-11)	6.5 (1-13)	21 (5-50)	6473 (2133-7600)	1 (12.5 %)	1 (12.5 %)	38 (34-40)	2 (25 %)	0 (0 %)
**MCD**	3	2 (66.6 %)	9.3 (0.5-10.1)	10 (1-17)	42 (15-65)	3178 (1225-3400)	0 (0 %)	2 (66.6 %)	39 (36-40)	2 (66.6 %)	1 (33.3 %)
**Other**	2	1	3-11.6	3-12	47-106	459-5461	0 (0 %)	0 (0 %)	38-40	1	1

*MesGN m*esangial proliferative glomerulopathy, *FSGS* focal segmental glomerulosclerosis, *DMS* diffuse mesangial sclerosis, *MCD* minimal change disease

Data are presented as median (range) or number (percentage)

### Histological findings

Renal histology was obtained at 1–17 (median 6) months, of which 35 had a percutaneous renal biopsy only; one had nephrectomy only, and 13 had both. The histological categories of diagnoses were as follows (based on published morphological criteria as available in standard texts): 13 Mesangial proliferative glomerulonephritis (MesGN), 12 Focal segmental glomerulosclerosis (FSGS), 11 Finnish nephropathy type changes, eight Diffuse Mesangial Sclerosis (DMS), three Minimal change disease (MCD) and one each for Dense Deposit Disease (DDD) and Membranous nephropathy. Histological examination was considered adequate in the majority of samples with 4–106 (median 30) glomeruli per biopsy and less than five and ten glomeruli in one and six biopsies, respectively (Table 1).

### Long-term outcome

Patient and renal survival was 73 % (32 children) and 43 % (19 children), respectively, at follow-up duration of 5–222 (median 73) months with five lost to follow-up (follow-up less than three months duration; three CNS and two INS). Patient survival for CNS and INS was 64 % (18 children) and 88 % (14 children), respectively. Renal survival for CNS and INS was 29 % (eight children; four MesGN, two FSGS and two Finnish) and 69 % (11 children; five DMS, five FSGS and one Finnish), respectively. Two children died due to haemorrhagic complications of the renal biopsy itself.

At the last follow-up at 5–222 (median 73) months; eight children were in remission (six complete remission and two partial remission), seven persistent NS, 14 were transplanted (three had been re-transplanted and six had functioning renal allografts), ten receiving dialysis, one with chronic kidney disease, with 12 mortalities, and five lost to follow-up. Five of those who died received renal replacement therapy: three received dialysis; one received both dialysis and transplantation, and one child received a pre-emptive renal transplant. The underlying cause of death was infection (peritonitis or sepsis in four children), haemorrhagic complications of renal biopsy (*N* = 2), intracranial haemorrhage associated with hypertension and anticoagulation therapy (*N* = 1), gastrointestinal haemorrhage (*N* = 1). Details of the underlying cause were not available in four children.

### Histology-associated outcome

#### Finnish

At last follow-up of the 11 patients with Finnish-type histological changes at 14–79 (median 44) months, seven children were alive, three deceased and one lost to follow-up. Four had undergone nephrectomies (three bilateral and one unilateral), five required dialysis at 2–11 (median 9) months of age. Four of those dialysed received a subsequent renal transplantation. One child (with Finnish-type histological features on needle biopsy) went into spontaneous remission at seven months of age, although genetic testing was not performed. On the last follow-up at four years of age, he remained in remission with normal renal function. (Table 2)

**Table 2:** Outcome according to histology. All transplants were preceded by dialysis apart from one child with Mesangioproliferative histology who received pre-emptive transplantation

	n	Remission	Dialysis	Renal transplantation	Mortality rate	Persistent NS	Lost to follow-up	Follow-up (months) (range)
MesGN	13	3 (23 %)	4 (31 %)	3 (8 %)	2 (15 %)	2 (15 %)	1 (8 %)	155(51-180)
**FSGS**	12	2 (17 %)	6(50 %)	3 (25 %)	2 (17 %)	2 (17 %)	1 (8 %)	26 (5-216)
**Finnish**	11	1(9 %)	5 (55.5 %)	4 (44 %)	3 (27 %)	1 (9 %)	2 (18 %)	44 (14-79)
**DMS**	8	0 (0 %)	3 (37.5 %)	2 (25 %)	4 (50 %)	1 (13 %)	1 (13 %)	55 (19-195)
**MCD**	3	1 (33 %)	1 (33.3 %)	1 (33 %)	1 (33 %)	1 (33 %)	0 (0 %)	192(118-222)
**Other**	2	1 (50 %)	1 (50 %)	1 (50 %)	0 (0 %)	0 (0 %)	0 (0 %)	44-48
**Total**	49	8 (16 %)	20 (41 %)	14 (29 %)	12 (24 %)	7 (14 %)	5 (10 %)	73 (5-222)

*MesGN* mesangial proliferative glomerulopathy, *FSGS* focal segmental glomerulosclerosis, *DMS* diffuse mesangial sclerosis, *MCD* minimal change disease, *NS* nephrotic syndrome

#### MesGN

Thirteen children had a histopathological diagnosis of MesGN. At the last follow-up ten children were alive, two had died and one lost to follow-up, with three children achieving remission. Four children required dialysis, and two of these subsequently received a renal transplant (one of whom was pre-emptively transplanted). Two children remained with persistent NS.

#### FSGS

Of the 12 patients with FSGS, nine were alive at 5–216 (median 26) months, two of whom achieved remission, five required dialysis 3–147 (median 46) and three of those received a renal transplant (Table 2).

#### DMS

For the eight patients with DMS, four were alive at the last follow-up at 19–195 (median 55) months, while four had died at 1–16 (median 6) months and one patient was lost to follow-up. Three children required dialysis at 7, 14, and 98 months of age. Two of those dialysed had subsequent renal transplantation, and one of them had nephrectomy at the age of 17 months of age (Table 2).

#### MCD

Two children with MCD were alive at 118–222 months follow-up. One achieved remission; one had persistent NS, while the third child required dialysis (at 12 months of age) followed by transplantation at 35 months of age, which was complicated by gastrointestinal haemorrhage; he subsequently died (Table 2). The child who achieved remission was treated with prednisolone and cyclophosphamide.

The case summaries of two children with an uncommon histological diagnosis for this young age are described as follows:

1. Dense Deposit Disease: a three-month-old girl presented with a clinical nephritic-nephrotic syndrome, hypocomplemenaemia (low C3), and acute kidney injury. The biopsy showed diffusely abnormal glomeruli, with 80 % showing cellular or fibrocellular crescents and MPGN morphological pattern. There was strong staining with C3 along glomerular capillary walls and in globules in the mesangium. Electron microscopy confirmed dense deposit disease. She was treated with plasma exchange and cyclophosphamide, with a progressive worsening of renal function (plasma creatinine of 440 μmol/l). She was dialysed and eventually transplanted without recurrence of DDD in her renal transplant.
2. Membranous nephropathy: an 11 month-old-boy, with a family history of membranous nephropathy (two uncles, who presented at three and ten years of age, and a great-uncle, who was diagnosed at 67 years of age) developed steroid-resistant NS with hypertension and microscopic haematuria [18]. His renal biopsy showed 47 diffusely abnormal glomeruli, with segmental mesangial expansion and marked thickening of the glomerular capillary walls. Immunohistochemical staining revealed diffuse capillary loop granular deposition of IgG, C1q and C3 and electron microscopy confirmed membranous nephropathy. He was commenced on mycophenolate mofetil (MMF), ACE inhibitors, and ARB with a slow but persistent improvement. At the age of ten years, he has had two prolonged relapses, which eventually resolved with increased immunosuppression, and he has normal renal function.

### Immunouppression

Corticosteroids were used in seven children (Table 1) and other immunsuppressive agents in three children. As detailed above, one child with MCD and the child with DDD received a course of oral cyclophosphamide, while the child with membranous nephropathy was treated with MMF.

### Nephrectomies

Thirteen children had nephrectomies following renal biopsies (ten bilateral and three unilateral) performed at 2–147 (median 14) months. The result of the histological examination of the resected kidneys showed a similar histological diagnosis in eight patients (seven with Finnish type changes and one with DMS), while four cases demonstrated extensive changes of ESKD with no further specific comment possible (one nephrectomy specimen was not available for re-analysis). The clinical indication for performing nephrectomies prior to ESKD management was severe nephrotic syndrome in order to minimise protein losses, reduce the risk of thrombosis, and enable an early diagnosis of potential recurrence of NS [19]. Dialysis (mainly peritoneal dialysis) was performed in 13 nephrectomised children and in seven other children.

One patient had two renal biopsies; the initial biopsy at four months of age suggested Finnish type changes, and the second biopsy two months later showed similarly dilated tubules with additional focal glomerulosclerosis without any mutation detected on his genetic studies. He died at 8 months of age because of sepsis, thromboembolism, gastrointestinal haemorrhage, and chronic kidney disease.

### Extra-renal manifestations

In addition to clinical NS, ten children had major gastro-intestinal disorders; seven had neurological complications, and five had congenital heart disease. Non-infectious chronic diarrhoea was the key symptom in six of the ten cases with gastro-intestinal disorders, with severe weight loss and the temporary need for total parenteral nutrition in two patients. These children underwent intestinal biopsies, which showed normal mucosa in three cases and moderate partial villous atrophy in the other three cases, one of which had an increase of inflammatory cell density with some eosinophils. The other gastro-intestinal complications were relapsing pancreatitis in two patients and hepatomegaly in two patients (with liver biopsies revealing a centrilobular hepatocyte necrosis in one case and normal in the other case). Neurologic disorders were mainly represented by severe developmental delay in seven children associated with microcephaly in three cases and seizures in four children. One child had hydrocephalus, which required ventriculoperitoneal shunt insertion. Two patients had mild dysmorphic features which did not fit with an identifiable syndrome; one of them had cloudy cornea. Congenital heart disease was observed in two children with Finnish-type histology (one had pulmonary stenosis and one had patent ductus arteriosus), while one child with MCD had an atrial septal defect.

### Genetic studies

Genetic results were available for 14 children only due to the historical nature of this cohort, with nine having causative mutations identified (Table 3). All of those with identified causative mutations presented before four months of age and did not achieve remission. Six required renal replacement therapy (RRT), one died, and one was lost to follow-up. In only two children was a heterozygote NPHS1 mutation detected (c.1868G > T; p.C623F with Finnish-type histological changes and c.1223G > A;p.R408Q with DMS type histology). Both of them required dialysis and transplantation. It is possible that the second mutation was not identified. The child with a homozygous mutation in ITGA3 has been reported previously [20]. The child with the homozygous PLCE1 mutation also had an atrial septal defect and right atrial thrombectomy. She had chronic recurrent pancreatitis and developed new onset of diabetes after transplantation (NODAT) requiring insulin. The histological diagnosis and outcome of the five children who had genetic testing performed but without identified causative mutations are summarized in Table 4. All patients with causative NPHS1 mutations had at least one severe (nonsense or splice site) mutation, except for the patient with the homozygous R367C mutation, which has been previously shown to cause aberrant trafficking of nephrin [21].

**Table 3:** Causative mutations identified

Pathology	Age at presentation (months)	Mutation found	Age at biopsy (months)	Outcome
FSGS	0.1	NPHS1: c.3478C>T; p.Arg1160* (Homozygous)	16	Nephrectomy and Pd at 46 months and transplantation at 64 month
Finnish	0.1	NPHS1: c.1758-8_1785del deletion of 36 bp including obligate splice site (Homozygous) plus NPHS2: c.Arg229Gln (h)	7	Nephrectomy at 11 months and transplantation at 14 months
MesGN	0.3	NPHS1: c.1868G>T; p.C623F and c.2335-1G>A (compound heterozygous)	1	Nephrectomy and PD at 4,5 years and transplantation and transplantation at 5.5 years of age
FSGS-MCD	0.3	PLCE1: c.961C>T; p.Arg321* (Homozygous)	4	PD at 2 years and transplantation at 3 years of age
Finnish	1	NPHS1: c.2227C>T; p.Arg743Cys and c.3442C>T; p.Gln1148* (compound heterozgous)	nephrectomy at 6 months	Haemodialysis at 6 years and transplantation at 6.5 years of age
Finnish	1.6	NPHS1: c.1099C>T; p.Arg367Cys (Homozygous)	3	Nephrectomy and dialysis at 7 months and transplantation at 24 months
Finnish	1.9	NPHS1: c.2227C>T; p.Arg743Cys and c.3481+1G>T (compound heterozygous)	7	Dialysis at 9 months of age
FSGS	4.3	ITGA3: c.1883G>C, p.Arg628Pro (Homozygous)	5	Died after commencing dialysis
MCD-FSGS	4	NPHS1 c.614_621delinsTT p.Thr205_Arg207delinsIle and c.2928G>T, p.Arg976Ser (compound heterozygous)	5	Nephrectomy, dialysis followed by transplantation at 12 years of age

*MesGN m*esangial proliferative glomerulopathy, *FSGS* focal segmental glomerulosclerosis, *DMS* diffuse mesangial sclerosis, *MCD* minimal change disease

**Table 4:** Outcome and findings in children without identified causative mutations

Pathology	Age at presentation(months)	Mutation found	Age at biopsy (months)	Outcome
Finish	0.3	Hetrozygous NPHS1 variant (c.320C>T;p.Ala107Val)	7	Dialysis at 10 months of age
FSGS	1	None	2	Dilaysis at 3 months of gae and died from sepsis at 9 months of age
MesGN	5.5	None	5	Remission
DMS	8.6	Heterozygote NPHS1 variant (c.1223G>A;p.Arg408Gln)	10	Dialysis at 15 months of age
Membranous	11.6	None	12	Remssion

*MesGN m*esangial proliferative glomerulopathy, *FSGS* focal segmental glomerulosclerosis, *DMS* diffuse mesangial sclerosis, *MCD* minimal change disease

## Discussion

The findings of the present study demonstrate that NS presenting in the first year of life is caused by heterogeneous glomerular diseases and that the histological diagnosis, whilst providing prognostic information, does not predict outcome in all cases. Genetic results were available only in a minority of patients, but none of the children with identified causative mutations achieved remission and all required RRT. Of course, genetic testing in such a retrospective study is highly likely to have included preselected cases in whom biopsy findings would suggest a high yield of positive mutations.

All children with Finnish-type histopathological changes presented before five months of age. Similarly, the majority of children with DMS presented early within the first six months of life. However, some children with FSGS, MesGN, MCD, and DDD also presented early, even within the first three months of age as also described by others [6, 7].

Histopathological findings cannot predict outcome with certainty since disease course varies among aetiological group. For example, one child with apparent Finnish-type histological features on initial needle biopsy, achieved remission. Remission in children with primary CNS associated with Finnish histopathology, DMS [15] or other patterns of histopathological changes is uncommon but has been reported previously [17]. This is different from the subgroup of infantile NS secondary to infections which carries a good prognosis with improvement as the underlying disease is treated [22].

Around one sixth of the children had a positive family history of an affected sibling and there was history of parental consanguinity in 31 %. Sixty-four percent of tested children had an identifiable genetic cause with homozygous or compound heterozygous mutations in NPHS1 (six patients), PLCE1 (one patient) or ITGA3 (one patient). All those presented early and failed to achieve remission. This is similar to previous studies where mutations were found to be associated with an earlier onset of disease and worse renal outcomes [7, 23]. However, milder cases were also reported to be associated with either two NPHS1 mutations in the cytoplasmic tail or two missense mutations in the extracellular domain, including at least one that preserved structure and function [7]. One child with a histological diagnosis of FSGS had underlying PLCE1 mutations. Although PLCE1 has been primarily associated with DMS it has been previously described to be mutated in a non-negligible proportion of FSGS cases without NPHS2 mutations [24].

None of the children with mutations responded to treatment or achieved remission, which is in keeping with the recommendation that most patients with genetic SRNS do not benefit from immunosuppression with rapid progression to ESKD [25, 26].

Six children suffered from considerable diarrhoea, which has been previously described in infantile nephrotic syndrome and may be explained by infection [2] or as a result of losing plasma proteins in the intestine due to intestinal oedema [27]. Two subjects suffered from chronic relapsing pancreatitis with one death associated with this complication. Acute pancreatitis can be related to renal venous and inferior vena cava thrombosis, which is a known complication of NS [28]. We observed developmental delay in seven children, which was associated with microcephaly in three of them. However, none of them had other features of Galloway-Mowat syndrome such as hypotonia and hiatus hernia [29]. This may be related to the often prolonged hospitalizations, complicated by septic episodes and multiple intensive care stays and/or the severe protein loss with failure-to-thrive.

We had one girl who presented with CNS with a renal biopsy confirming DDD and resulting in rapid progression to ESKD. DDD has been reported as a cause of SRNS in young children [30, 31] and in infants [32] but not as a cause of CNS. We had one patient who presented at one year of age with NS and biopsy revealed membranous histopathology. He achieved remission which is similar to previous reports of membranous nephropathy as a rare cause of NS in children with benign outcome [33].

Historically, prior to the availability of genetic testing, percutaneous renal biopsies were performed to understand the histopathology and guide treatment with prognostic information. However, in our cohort, two children died as a result of haemorrhagic complications from the renal biopsy itself. Unfortunately, there were small numbers of patients in some of the subgroups (such as MCD) to be able to comment on overall prognosis, but unsurprisingly, only one of the Finnish type patients went into remission, and none of the DMS patients, who had the highest mortality rate of 50 %.

Children with identified causative mutations generally presented earlier and had poor renal outcome. The time taken to obtain genetic testing results is decreasing with improving technology. The availability of genetic testing varies from centre to centre and country to country. More importantly, reimbursement for these tests is highly variable, so that in some cases, testing can only be performed by research labs with undefined time spans for the availability of results. Thus, in some centres, it may not be possible to obtain a test or wait for the result within a time frame that would allow integration of the result in the clinical management. However, the general trend is for these tests to become increasingly available at low cost. If widespread genetic testing were available, for all infants presenting with nephrotic syndrome or SRNS in the first year of life, renal biopsy could be reserved for children with atypical presentations such as a mixed nephritic-nephrotic picture, hypocomplementaemia or in those in whom no causative mutations are identified.

## References

[1] Rivera A, Meleg-Smith S, Yosipiv I, El-Dahr S, Boineau F (2003). Diagnosis of congenital nephrotic syndrome: a clinical and a pathologic challenge, Pediatr Pathol. Mol Med.

[2] Habib R (1993). Nephrotic syndrome in the 1st year of life. Pediatr Nephrol.

[3] Patrakka J, Kestila M, Wartiovaara J, Ruotsalainen V, Tissari P, Lenkkeri U, Mannikko M, Visapaa I, Holmberg C, Rapola J, Tryggvason K, Jalanko H (2000). Congenital nephrotic syndrome (NPHS1): features resulting from different mutations in Finnish patients. Kidney Int.

[4] Habib R, Gubler MC, Antignac C, Loirat C, Gangnadoux MF (1990). Congenital or childhood nephrotic syndrome with diffuse mesangial sclerosis. Ann Pediatr (Paris).

[5] Ozen S, Tinaztepe K (1996). Diffuse mesangial sclerosis: a unique type of congenital and infantile nephrotic syndrome. Nephron.

[6] Kaneko K, Suzuki Y, Kiya K, Matsubara T, Fukuda Y, Yabuta K (1998). Minimal change lesion in congenital nephrotic syndrome. Two case reports and a review of the literature. Nephron.

[7] Machuca E, Benoit G, Nevo F, Tete MJ, Gribouval O, Pawtowski A, Brandstrom P, Loirat C, Niaudet P, Gubler MC, Antignac C (2010). Genotype-phenotype correlations in non-Finnish congenital nephrotic syndrome. J Am Soc Nephrol.

[8] Schoeb DS, Chernin G, Heeringa SF, Matejas V, Held S, Vega-Warner V, Bockenhauer D, Vlangos CN, Moorani KN, Neuhaus TJ, Kari JA, MacDonald J, Saisawat P, Ashraf S, Ovunc B, Zenker M, Hildebrandt F (2010). Nineteen novel NPHS1 mutations in a worldwide cohort of patients with congenital nephrotic syndrome (CNS). Nephrol Dial Transplant.

[9] Schumacher V, Scharer K, Wuhl E, Altrogge H, Bonzel KE, Guschmann M, Neuhaus TJ, Pollastro RM, Kuwertz-Broking E, Bulla M, Tondera AM, Mundel P, Helmchen U, Waldherr R, Weirich A, Royer-Pokora B (1998). Spectrum of early onset nephrotic syndrome associated with WT1 missense mutations. Kidney Int.

[10] Gbadegesin R, Hinkes BG, Hoskins BE, Vlangos CN, Heeringa SF, Liu J, Loirat C, Ozaltin F, Hashmi S, Ulmer F, Cleper R, Ettenger R, Antignac C, Wiggins RC, Zenker M, Hildebrandt F (2008). Mutations in PLCE1 are a major cause of isolated diffuse mesangial sclerosis (IDMS). Nephrol Dial Transplant.

[11] Chen YM, Kikkawa Y, Miner JH (2011). A missense LAMB2 mutation causes congenital nephrotic syndrome by impairing laminin secretion. J Am Soc Nephrol.

[12] Hinkes BG, Mucha B, Vlangos CN, Gbadegesin R, Liu J, Hasselbacher K, Hangan D, Ozaltin F, Zenker M, Hildebrandt F (2007). Nephrotic syndrome in the first year of life: two thirds of cases are caused by mutations in 4 genes (NPHS1, NPHS2, WT1, and LAMB2). Pediatrics.

[13] Roussel B, Pinon JM, Birembaut P, Rullier J, Pennaforte F (1987). Congenital nephrotic syndrome associated with congenital toxoplasmosis. Arch Fr Pediatr.

[14] Rahman H, Begum A, Jahan S, Muinuddin G, Hossain MM (2008). Congenital nephrotic syndrome, an uncommon presentation of cytomegalovirus infection. Mymensingh Med J.

[15] Xiao HJ, Liu JC, Zhong XH (2011). Congenital syphillis presenting congenital nephrotic syndrome in two children and related data review. Beijing Da Xue Xue Bao.

[16] Debiec H, Nauta J, Coulet F, van der Burg M, Guigonis V, de Heer E, Soubrier F, Janssen F, Ronco P (2004). Role of truncating mutations in MME gene in fetomaternal alloimmunisation and antenatal glomerulopathies. Lancet.

[17] Sreedharan R, Bockenhauer D (2005). Congenital nephrotic syndrome responsive to angiotensin-converting enzyme inhibition. Pediatr Nephrol.

[18] Bockenhauer D, Debiec H, Sebire N, Barratt M, Warwicker P, Ronco P, Kleta R (2008). Familial membranous nephropathy: an X-linked genetic susceptibility?. Nephron Clin Pract.

[19] Holmberg C, Antikainen M, Ronnholm K, Ala HM, Jalanko H (1995). Management of congenital nephrotic syndrome of the Finnish type. Pediatr Nephrol.

[20] Has C, Sparta G, Kiritsi D, Weibel L, Moeller A, Vega-Warner V, Waters A, He Y, Anikster Y, Esser P, Straub BK, Hausser I, Bockenhauer D, Dekel B, Hildebrandt F, Bruckner-Tuderman L, Laube GF (2012). Integrin alpha3 mutations with kidney, lung, and skin disease. N Engl J Med.

[21] Liu XL, Done SC, Yan K, Kilpelainen P, Pikkarainen T, Tryggvason K (2004). Defective trafficking of nephrin missense mutants rescued by a chemical chaperone. J Am Soc Nephrol.

[22] Kim JJ, Clothier J, Sebire NJ, Milford DV, Moghal N, Trompeter RS (2011). Nephrotic syndrome in infancy can spontaneously resolve. Pediatr Nephrol.

[23] Santin S, Bullich G, Tazon-Vega B, Garcia-Maset R, Gimenez I, Silva I, Ruiz P, Ballarin J, Torra R, Ars E (2011). Clinical utility of genetic testing in children and adults with steroid-resistant nephrotic syndrome. Clin J Am Soc Nephrol.

[24] Boyer O, Benoit G, Gribouval O, Nevo F, Pawtowski A, Bilge I, Bircan Z, Deschenes G, Guay-Woodford LM, Hall M, Macher MA, Soulami K, Stefanidis CJ, Weiss R, Loirat C, Gubler MC, Antignac C (2010). Mutational analysis of the PLCE1 gene in steroid resistant nephrotic syndrome. J Med Genet.

[25] Buscher AK, Kranz B, Buscher R, Hildebrandt F, Dworniczak B, Pennekamp P, Kuwertz-Broking E, Wingen AM, John U, Kemper M, Monnens L, Hoyer PF, Weber S, Konrad M (2010). Immunosuppression and renal outcome in congenital and pediatric steroid-resistant nephrotic syndrome. Clin J Am Soc Nephrol.

[26] Kari JA, El-Desoky SM, Gari M, Malik K, Vega-Warner V, Lovric S, Bockenhauer D (2013). Steroid-resistant nephrotic syndrome: impact of genetic testing. Ann Saudi Med.

[27] Kluthe R, Liem HH, Nussle D, Barandun S (1963). Intestinal plasma protein loss (protein diarrhea) in the nephrotic syndrome. Klin Wochenschr.

[28] Ma SK, Kim SW, Kim NH, Choi KC (2002). Renal vein and inferior vena cava thrombosis associated with acute pancreatitis. Nephron.

[29] Cohen AH, Turner MC (1994). Kidney in Galloway-Mowat syndrome: clinical spectrum with description of pathology. Kidney Int.

[30] Cansick JC, Lennon R, Cummins CL, Howie AJ, McGraw ME, Saleem MA, Tizard EJ, Hulton SA, Milford DV, Taylor CM (2004). Prognosis, treatment and outcome of childhood mesangiocapillary (membranoproliferative) glomerulonephritis. Nephrol Dial Transplant.

[31] Lu DF, Moon M, Lanning LD, McCarthy AM, Smith RJ (2012). Clinical features and outcomes of 98 children and adults with dense deposit disease. Pediatr Nephrol.

[32] Kari JA, Bamashmous H, Lingawi S, Al-Sabban E, Akhtar M (2001). Infantile nephrotic syndrome and congenital glaucoma. Pediatr Nephrol.

[33] Obana M, Nakanishi K, Sako M, Yata N, Nozu K, Tanaka R, Iijima K, Yoshikawa N (2006). Segmental membranous glomerulonephritis in children: comparison with global membranous glomerulonephritis. Clin J Am Soc Nephrol.

